# Screening properties of trend tests in genetic association studies

**DOI:** 10.1038/s41598-023-35929-4

**Published:** 2023-06-05

**Authors:** Zhenzhen Jiang, Hongping Guo, Jinjuan Wang

**Affiliations:** 1 Academy of Mathematics and Systems Science, Chinese Academy of Sciences, Beijing, 100190 People’s Republic of China; 2 University of Chinese Academy of Sciences, Beijing, 100049 People’s Republic of China; 3 School of Mathematics and Statistics, Hubei Normal University, Huangshi, 435002 People’s Republic of China; 4 School of Mathematics and Statistics, Beijing Institute of Technology, Beijing, 100081 People’s Republic of China

**Keywords:** Genetics, Medical research

## Abstract

In genome-wide association study, extracting disease-associated genetic variants among millions of single nucleotide polymorphisms is of great importance. When the response is a binary variable, the Cochran-Armitage trend tests and associated MAX test are among the most widely used methods for association analysis. However, the theoretical guarantees for applying these methods to variable screening have not been built. To fill this gap, we propose screening procedures based on adjusted versions of these methods and prove their sure screening properties and ranking consistency properties. Extensive simulations are conducted to compare the performances of different screening procedures and demonstrate the robustness and efficiency of MAX test-based screening procedure. A case study on a dataset of type 1 diabetes further verifies their effectiveness.

## Introduction

With the development of high throughput sequencing techniques, hundreds of thousands of single nucleotide polymorphisms (SNPs) in the genome are recorded, which enables researchers to investigate and treat diseases from the perspective of genetic variants. To identify the disease-related genes or genetic markers among all these SNPs, genome-wide association study (GWAS) is a widely used strategy. Up to now, more than one hundred thousands of SNPs have been identified to be related to many traits^1–7^.

The commonly used GWAS tests the association between the phenotype and each SNP sequentially, obtains a series of test statistics or *p*-values, and selects the associated SNPs by comparing these statistics or *p*-values with a given threshold. When the phenotype is binary, Cochran-Armitage trend test (CATT)^8^ is always used to detect the associated SNPs. It has been shown that when the underlying genetic model is known, where the commonly used ones are recessive, additive or dominant models, CATT has an optimal form^9,10^. However, the true genetic models are always unknown and may be very complicated. For the sake of robustness, an omnibus test called MAX is proposed^11,12^, which uses the maximum of CATTs under different genetic models as a measure for association. The asymptotical distribution of MAX is given in the work of Zheng et al.^13^. Since its being raised, MAX has been widely used and investigated. Li et al.^14^ introduced a selection procedure based on the rank of MAX. Kim et al.^15^ proposed a SNP selection method based on MAX and a penalized support vector machine strategy.

Though CATTs and MAX have concise forms and are extensively used, theoretical properties for the applications of CATTs and MAX to GWAS have not been investigated. To control false discovery rate (FDR) in GWAS, Bonferroni correction strategy and FDR control procedures, such as Benjamini–Hochberg procedure, are two widely used strategies. But they both assume that all the SNPs are independent, which certainly is improperly since linkage disequilibrium usually exists among SNPs and may lead to omission on related SNPs. Considering these drawbacks, feature screening methods are sensible alternatives. Rather than select the associated SNPs directly, feature screening approaches aim to eliminate most of the irrelevant SNPs at first. After a screening procedure, there remains only a small amount of SNPs and researchers can concentrate on these remaining SNPs, which can save much time and work.

In the last few years, feature screening methods have been proposed for various situations. Fan and Lv^16^ first proposed a screening method called the sure independence screening approach for Gaussian response and predictors under linear regressions. Since then, sure screening property, which retains all the important predictors with high probability as the sample size goes into infinity, has been regarded as a feature screening criterion. Many screening procedures have been developed for diverse models, such as the generalized linear model^17^ and additive model^18^ among others. Although many procedures can be directly applied to GWAS with corresponding models and data types, only PC-SIS, proposed in the work of Huang et al.^19^, is applicable to the considered situation where both the outcome and predictors are categorical. However, PC-SIS does not take the information on genetic model into consideration. Just as mentioned above, CATTs and MAX test consider this information in the association analysis. But their screening properties have not been studied yet. To fill this gap, we propose feature screening methods based on CATTs in different genetic models and MAX test, and investigate their sure screening and rank consistency properties.

The rest of paper is organised as follows. In “Trend test”, we briefly describe the trend tests which can be used to evaluate the relationship between a binary variable and a genotype variable. “Independence screening procedure” introduces the independence screening procedures based on the adjusted trend test statistics, and presents sure screening and ranking consistency properties. Simulation studies are conducted in “Simulation studies” . And a case study on type 1 diabetes is demonstrated in “Application to a real dataset”. A conclusion for this work is presented in “Conclusion”. All proofs of theorems are provided in the Supplemental Materials.

## Trend test

CATT evaluates the association between a binary variable and a SNP, and is widely used in case-control genetic data analysis. Compared with Pearson chi-square test, it makes use of the underlying genetic model. Its specific form is as follows. Suppose *r* cases and *s* controls are enrolled in the study. For a given SNP, the genotypes can be expressed as aa, Aa and AA, respectively, with A being a high risk candidate allele. In the sample of cases, the counts of aa, Aa and AA are $r_0,~r_1$ and $r_2$, respectively. And the corresponding counts in the control samples are $s_0,~s_1$ and $s_2$. Thus we have $r = r_0+r_1+r_2, ~s=s_0+s_1+s_2$. Denote $n=r+s$ and $n_i = r_i+s_i$ for $i = 0,1,2$. All these counts are displayed in Table 1. Then CATT can be written as

1 $$\begin{aligned} Z =\frac{\sqrt{n}\sum \limits _{i=0}^{2}X_i(sr_i-rs_i)}{\sqrt{rs\big [n\sum \limits _{i=0}^{2}X_i^2n_i- (\sum \limits _{i=0}^{2}X_in_i)^2\big ]}}, \end{aligned}$$

where $(X_0,X_1,X_2)$ is a pre-defined genotype score vector. Note that the optimal score vector for CATT varies across different genetic models. Specifically, for the commonly encountered recessive genetic model, additive genetic model and dominant genetic model, the optimal genotype score vectors are (0, 0, 1), $(0,\frac{1}{2},1)$ and (0, 1, 1), respectively. And the respective corresponding CATT can be denoted as $Z_{0}, Z_{\frac{1}{2}}$ and $Z_{1}$. Under the null hypothesis of no association, these three CATTs above are asymptotically normally distributed as *N*(0, 1).

**Table 1:** Genotype distribution in sample.

	aa	Aa	AA	Total
Cases	$r_0$	$r_1$	$r_2$	*r*
Controls	$s_0$	$s_1$	$s_2$	*s*
Total	$n_0$	$n_1$	$n_2$	*n*

However, in practice, the true genetic model is unknown. Thus none of $Z_{0}, Z_{\frac{1}{2}}$ and $Z_{1}$ is robust in all situations. To tackle this issue, the statistic MAX is proposed as

2 $$\begin{aligned} Z_{max} = \max \{|Z_{0}|, |Z_{\frac{1}{2}}|, |Z_{1}|\}. \end{aligned}$$

By using the maximum of absolute values of $Z_{0}, Z_{\frac{1}{2}}$ and $Z_{1}$, $Z_{max}$ obtains robustness under diverse situations.

## Independence screening procedure

### Screening procedure

CATTs and MAX test are designed for testing the relationship between a binary response and a SNP variable. We apply them to feature screening task and display their properties.

Suppose $\textbf{G} = (G_1,G_2,\ldots ,G_m)^{\top }$ is a *m*-dimensional SNP vector and *Y* is a binary response which is 1 for a case sample and 0 for a control sample. Denote $P(Y=1) = p$ and $P(Y=0)=q$, where $p+q=1.$ Our aim is to identify the SNPs among all the *m* SNPs that are related with *Y*. In accordance with practice, each SNP takes value in $\{0,1,2\}$, corresponding to genotypes aa, Aa and AA, respectively.

For the *k*th $(k=1,2,\ldots ,m)$ predictor $G_k$, we set probabilities for case population as $p_{ik} = P(G_k = i, Y = 1), i =0,1,2$ and those for control population as $q_{ik} = P(G_k = i, Y=0), i =0,1,2$, which are displayed in Table 2. Note that $p_{0k}+p_{1k}+p_{2k} =p$ and $q_{0k}+q_{1k}+q_{2k}=q$ for each *k* in $\{1,2,\ldots ,m\}$. Denote $f_{ik} = p_{ik} + q_{ik}, i = 0,1,2, k= 1,2,\ldots ,m$. Then $f_{0k}+f_{1k}+f_{2k}=1, k= 1,2,\ldots ,m$.

**Table 2:** Genotype distribution in population.

	$G_k=0$	$G_k=1$	$G_k=2$	Tatal
$Y=1$	$p_{0k}$	$p_{1k}$	$p_{2k}$	*p*
$Y=0$	$q_{0k}$	$q_{1k}$	$q_{2k}$	*q*
Total	$f_{0k}$	$f_{1k}$	$f_{2k}$	1

Denote the pre-defined score vectors for the recessive, additive and dominant genetic model as $(X_{0,0},X_{1,0},X_{2,0})= (0,0,1)$, $(X_{0,\frac{1}{2}},X_{1,\frac{1}{2}},X_{2,\frac{1}{2}})= (0,\frac{1}{2},1),$ and $(X_{0,1},X_{1,1},X_{2,1})= (0,1,1)$, respectively. Then define four measures for the association relationship between $G_k(k = 1,2,\ldots ,m)$ and *Y* as

3 $$\begin{aligned} \begin{array}{lll} \omega _{j,k} = \frac{\sum \limits _{i=0}^{2}X_{i,j}(qp_{ik}-pq_{ik})}{\sqrt{pq\big [\sum \limits _{i=0}^{2}X_{i,j}^2f_{ik}- (\sum \limits _{i=0}^{2}X_{i,j}f_{ik})^2\big ]}}, \quad j = 0,\frac{1}{2},1; \quad k=1,2,\ldots ,m, \end{array} \end{aligned}$$

and

4 $$\begin{aligned} \begin{array}{lll} \nu _k = \max \{|\omega _{0,k}|,|\omega _{\frac{1}{2},k}|,|\omega _{1,k}|\}, \quad k=1,2,\ldots ,m. \end{array} \end{aligned}$$

It is obvious that when $G_k (k = 1,2,\ldots ,m)$ is independent of *Y*, $\omega _{j,k} = 0( j = 0,\frac{1}{2},1)$ and $\nu _k = 0$.

For $k \in \{1,2,\ldots , m\}$, let $\{(g_{lk},y_l),l = 1,2,\ldots ,n\}$ be *n* pairs of observations of $(G_k,Y)$. Denote ${{\varvec{r}}_k} = (r_{0k},r_{1k},r_{2k})^{\top },{{\varvec{s}}_k} = (s_{0k},s_{1k},s_{2k})^{\top },$ where $r_{ik} ~(i =0,1,2)$ are the counts of each genotype in case sample and $s_{ik}~(i =0,1,2)$ are the counts in control sample. Notice that $r_{0k}+r_{1k}+r_{2k} =r$ and $s_{0k}+s_{1k}+s_{2k}=s$. Denote $n_{ik}=r_{ik}+s_{ik}, i = 0,1,2$, then we have $n_{0k}+n_{1k}+n_{2k} =n$.

Given the above notations, the empirical estimators of $\omega _{0,k},\omega _{\frac{1}{2},k},\omega _{1,k}$, and $\nu _k$ for $k \in \{1,2,\ldots , m\}$ are

5 $$\begin{aligned} \begin{array}{lll} {\hat{\omega }}_{j,k}=\frac{\sum \limits _{i=0}^{2}X_{i,j}({\hat{q}}{\hat{p}}_{ik} -{\hat{p}}{\hat{q}}_{ik})}{\sqrt{{\hat{p}}{\hat{q}}\big [\sum \limits _{i=0}^{2}X_{i,j}^2{\hat{f}}_{ik}- (\sum \limits _{i=0}^{2}X_{i,j}{\hat{f}}_{ik})^2\big ]}}, \quad j = 0,\frac{1}{2},1, \end{array} \end{aligned}$$

and

6 $$\begin{aligned} \begin{array}{lll} {\hat{\nu }}_k = \max \{|{\hat{\omega }}_{0,k}|,|{\hat{\omega }}_{\frac{1}{2},k}|,|{\hat{\omega }}_{1,k}|\}, \end{array} \end{aligned}$$

where ${\hat{p}}_{ik},{\hat{q}}_{ik},{\hat{p}},{\hat{q}},{\hat{f}}_{ik}$ are the empirical estimators of $p_{ik},q_{ik},p,q,f_{ik}$, and can be estimated as

7 $$\begin{aligned} \begin{array}{lll} {\hat{p}}_{ik} &{}=&{} \frac{1}{n}\sum \limits _{l=1}^{n}I(G_{lk} = i,Y_{l} = 1) = \displaystyle {\frac{r_{ik}}{n}}\\ {\hat{q}}_{ik} &{}=&{} \frac{1}{n}\sum \limits _{l=1}^{n}I(G_{lk} = i,Y_{l} = 0) = \displaystyle {\frac{s_{ik}}{n}},\\ {\hat{p}} &{}=&{} \frac{1}{n}\sum \limits _{l=1}^{n}I(Y_{l} = 1) = \displaystyle {\frac{r}{n}},\\ {\hat{q}} &{}=&{} \frac{1}{n}\sum \limits _{l=1}^{n}I(Y_{l} = 0) = \displaystyle {\frac{s}{n}},\\ {\hat{f}}_{ik} &{}=&{} \frac{1}{n}\sum \limits _{l=1}^{n}I(G_{lk} = i) = \displaystyle {\frac{n_{ik}}{n}}.\\ \end{array} \end{aligned}$$

Plug them into the expression, ${\hat{\omega }}_{j,k}$ has the form

8 $$\begin{aligned} {\hat{\omega }}_{j,k} = \frac{\sum \limits _{i=0}^{2}X_{i,j}(sr_{ik}-rs_{ik})}{\sqrt{rs\big [n\sum \limits _{i=0}^{2}X_{i,j}^2n_{ik}- (\sum \limits _{i=0}^{2}X_{i,j}n_{ik})^2\big ]}}. \end{aligned}$$

Note that ${\hat{\omega }}_{j,k} = \frac{Z_{j,k}}{\sqrt{n}}$, where $Z_{0,k}, ~Z_{\frac{1}{2},k}$ and $Z_{1,k}$ are CATT statistics between $G_{k}$ and *Y* for the pre-defined score vector $(X_0,X_1,X_2)$ being (0, 0, 1),  $(0,\frac{1}{2},1)$ and (0, 1, 1), respectively. So ${\hat{\omega }}_{j,k}$ is an adjusted version of $Z_{j,k}$, whose value range is not effected by sample size. And ${\hat{\nu _k}}$ maintains the ranking result of $Z_{max,k}$ for each predictor. Large values of ${\hat{\nu _k}}$ indicate the existence of association between $G_k$ and *Y*. We denote ${\hat{\omega }}_{j,k}$ as aCATT and ${\hat{\nu _k}}$ as aMAX.

Assume that only a small part of SNPs are related with the response *Y*. We use aCATT $|{\hat{\omega }}_{j,k}|$s and aMAX ${\hat{\nu _k}}$s to identify their positions. The screening procedures based on $|{\hat{\omega }}_{0,k}|$s, $|{\hat{\omega }}_{\frac{1}{2},k}|$s, $|{\hat{\omega }}_{1,k}|$s and ${\hat{\nu _k}}$s are named as REC-SIS, ADD-SIS, DOM-SIS and MAX-SIS, respectively, where REC-SIS, ADD-SIS and DOM-SIS are collectively called as CATT-SIS.

### Screening properties

We call a SNP as an active SNP if it is associated with the response *Y*. Define different index sets of active SNPs based on different measures by

9 $$\begin{aligned} {\mathscr {A}}_j^*= & {} \{1\le k \le m:|\omega _{j,k}|>0\},\quad j = 0,\frac{1}{2},1, \end{aligned}$$

10 $$\begin{aligned} {\mathscr {A}}^*= & {} \{1\le k \le m:\nu _k>0\}. \end{aligned}$$

Their estimated truncated active index sets can be expressed as

11 $$\begin{aligned} \hat{{\mathscr {A}}}_j^*= & {} \{1\le k \le m:|{\hat{\omega }}_{j,k}|\ge c_0n^{-\tau }\},\quad j = 0,\frac{1}{2},1, \end{aligned}$$

12 $$\begin{aligned} \hat{{\mathscr {A}}}^*= & {} \{1\le k \le m:{\hat{\nu }}_k\ge c_0n^{-\tau }\}. \end{aligned}$$

where $c>0$ and $\tau >0$ are two pre-specified constants and satisfy some certain conditions.

Now we investigate the theoretical properties of the screening procedures of $\hat{{\mathscr {A}}}_{j}^*$ and $\hat{{\mathscr {A}}}^*$s. First list some conditions.

#### Condition 1

(C1): There exists constants $0< \zeta _{min} \le \zeta _{max} <1$ such that for $i = 0, 1,2$ and $k=1,2,\ldots ,m$, if $p_{ik}\ne 0 (q_{ik} \ne 0)$, then $p_{ik} \in (\zeta _{min},\zeta _{max}) (q_{ik} \in (\zeta _{min},\zeta _{max}))$.
(C2): $\min \limits _{k\in {\mathscr {A}}_{j}^{*}} \omega _{j,k} \ge 2c_0n^{-\tau }$ for $j = 0, \frac{1}{2}, 1$, where constant $c_0>0$ and $0\le \tau <\frac{1}{2}$.
(C3): $\min \limits _{k\in {\mathscr {A}}^{*}} \nu _k \ge 2c_0n^{-\tau }$, where constant $c_0>0$ and $0\le \tau <\frac{1}{2}$.
(C4): For given constants $c_0>0,0\le \tau <\frac{1}{2},$ and $\log (m)=o(n^{1-2\tau } \wedge n^{\frac{1}{2}})$ where $a\wedge b = \min \{a,b\}$, $\liminf \limits _{m\rightarrow \infty }(\min \limits _{k\in {\mathscr {A}}_j^{*}}\omega _{j,k} - \max \limits _{k\notin {\mathscr {A}}_j^{*}}\omega _{j,k}) >2c_0n^{-\tau }$ for $j = 0, \frac{1}{2}, 1$.
(C5): For given constants $c_0>0,0\le \tau <\frac{1}{2},$ and $\log (m)=o(n^{1-2\tau } \wedge n^{\frac{1}{2}})$ where $a\wedge b = \min \{a,b\}$, $\liminf \limits _{m\rightarrow \infty }(\min \limits _{k\in {\mathscr {A}}^{*}}\nu _{k} - \max \limits _{k\notin {\mathscr {A}}^{*}}\nu _{k}) >2c_0n^{-\tau }$.

Then we present the sure screening properties based on aCATT and aMAX in Theorem 1 and 2, whose proofs are shown in Supplemental Materials.

#### Theorem 1

(Sure Screening Property of CATT-SIS):

(i) *If Condition (C1) holds, then for* $j = 0, \frac{1}{2}$ *and 1 we have*
  13 $$\begin{aligned} \begin{array}{lll} P\big (\max \limits _{1\le k\le m} |{\hat{\omega }}_{j,k}-\omega _{j,k}| \ge c_0n^{-\tau }\big ) < O(m\exp \{-c_1n^{1-2\tau }-c_2n^{\frac{1}{2}}\}), \end{array} \end{aligned}$$
  *with* $c_1>0$ *and* $c_2>0$ *being two constants*.
(ii) *Furthermore, if both Conditions* (*C1*) and (*C2*) *are satisfied*, *for* $j = 0, \frac{1}{2}$ *and* 1 *we obtain that*
  14 $$\begin{aligned} P\big ({\mathscr {A}}_j^* \subseteq \hat{{\mathscr {A}}}_j^* \big ) \ge 1- O(\kappa \exp \{- c_1n^{1-2\tau }-c_2n^{\frac{1}{2}}\}), \end{aligned}$$
  *where* $\kappa$ *is the cardinality of* ${\mathscr {A}}_j^*$, *and* $c_1,c_2>0$ *are the same as those in inequality* (13).

#### Theorem 2

(Sure Screening Property for MAX-SIS):

(i) *If Condition* (*C1*) *holds, then we have*
  15 $$\begin{aligned} \begin{array}{lll} P\big (\max \limits _{1\le k\le m} |{\hat{\nu }}_{k}-\nu _k| \ge c_0n^{-\tau }\big ) < O(m\exp \{-c_3n^{1-2\tau }-c_4n^{\frac{1}{2}}\}), \end{array} \end{aligned}$$
  *where* $c_3>0$ *and* $c_4>0$ *are two constants*.
(ii) *Furthermore, if both Conditions* (*C1*) *and* (*C3*) *are satisfied*, *we have that*
  16 $$\begin{aligned} P\big ({\mathscr {A}}^* \subseteq \hat{{\mathscr {A}}}^* \big ) \ge 1- O(\kappa \exp \{- c_3n^{1-2\tau }-c_4n^{\frac{1}{2}}\}), \end{aligned}$$
  *where* $\kappa$ *is the cardinality of* ${\mathscr {A}}^*$, *and* $c_3,c_4>0$ *are the same as those in inequality* (15).

Theorems 1 and 2 show that the screening procedures have satisfying performances with regard to selecting significant SNPs. They also possess ranking consistency property, which are shown below.

#### Theorem 3

(Ranking Consistency Property for CATT-SIS): *Suppose Conditions* (*C1*) *and* (*C4*) *are satisfied, then for* $j = 0, \frac{1}{2}$ *and* 1, *it follows that*

17 $$\begin{aligned} \liminf \limits _{n\rightarrow \infty }\big \{\min \limits _{k \in {\mathscr {A}}_j^* } |{\hat{\omega }}_{j,k}| - \max \limits _{k \notin {\mathscr {A}}_j^*} |{\hat{\omega }}_{j,k}| \big \} \ge 0,\quad a.s. \end{aligned}$$

#### Theorem 4

(Ranking Consistency Property for MAX-SIS) *Suppose Condition* (*C1*) *and* (*C5*) *are satisfied, then it follows that*

18 $$\begin{aligned} \liminf \limits _{n\rightarrow \infty }\big \{\min \limits _{k \in {\mathscr {A}}^* } {\hat{\nu }}_k - \max \limits _{k \notin {\mathscr {A}}^*} {\hat{\nu }}_k \big \} \ge 0,\quad a.s. \end{aligned}$$

In practice, *c* and $\tau$ are hard to be determined to satisfy the condition that the estimated truncated active index sets contain the corresponding active index sets. So it is common to select SNPs corresponding to the first *d* largest statistic values as related SNPs, where *d* is a pre-defined constant. That is, the respective estimated active index sets have the following forms

$$\begin{aligned} \hat{{\mathscr {A}}}_{j,d}^* = \{1\le k \le m:|{\hat{\omega }}_{j,k}| \text { is among the first}\, d \,\text {largest statistics}\}, \end{aligned}$$

and

$$\begin{aligned} \hat{{\mathscr {A}}}_{d}^* = \{1\le k \le m:{\hat{\nu }}_k \text { is among the first}\, d \,\text {largest statistics}\}. \end{aligned}$$

We now explain why we determine the index sets corresponding to the first *d* largest statistics as active index sets. Take MAX-SIS for example. Given *c* and $\tau$, the cardinality of $\hat{{\mathscr {A}}}^*$ is determined, which is denoted as $d_0$. According to Theorem 4, MAX-SIS possesses ranking consistency property. Provided Conditions (C1) and (C5) are satisfied, we have $\hat{{\mathscr {A}}}^* \subseteq \hat{{\mathscr {A}}}_{d}^*$ if $d\ge d_0$. This indicates that all active predictors are all included in $\hat{{\mathscr {A}}}_{d}^*$. Note that $P({\mathscr {A}}^* \subseteq \hat{{\mathscr {A}}}_{d}^*)$ is nondecreasing in *d*. As long as $d\ge d_0$, we have $P({\mathscr {A}}^* \subseteq \hat{{\mathscr {A}}}_{d}^*) \ge P({\mathscr {A}}^* \subseteq \hat{{\mathscr {A}}}^*) \ge 1- O(\kappa \exp \{- c_3n^{1-2\tau }-c_4n^{\frac{1}{2}}\})$ based on Theorem 2 (ii). Therefore, estimating the active index set based on an index set corresponding to the first *d* largest statistics is reasonable.

## Simulation studies

**Figure 1:**
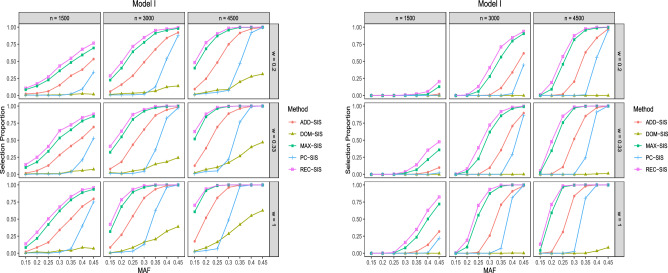
Selection proportions of different methods in Model I. The left subplot is for ${\mathcal {P}}^1_s$ among 500 repetitions. The right subplot is for ${\mathcal {P}}_a$ among 500 repetitions.

**Figure 2:**
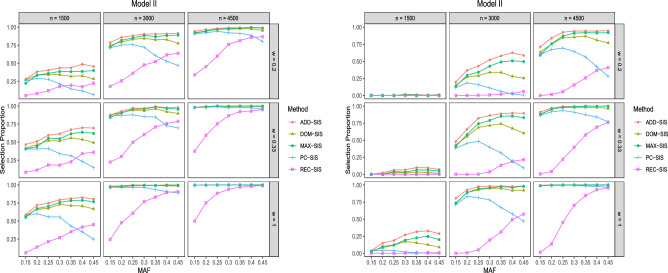
Selection proportions of different methods in Model II. The left subplot is for ${\mathcal {P}}^1_s$ among 500 repetitions. The right subplot is for ${\mathcal {P}}_a$ among 500 repetitions.

**Figure 3:**
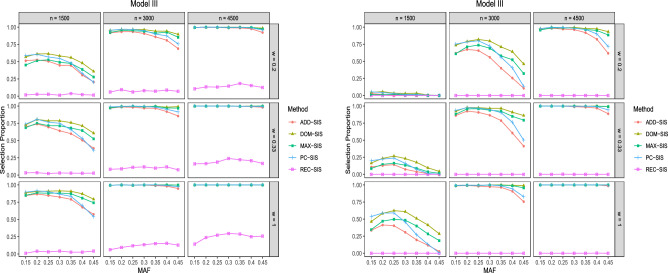
Selection proportions of different methods in Model III. The left subplot is for ${\mathcal {P}}^1_s$ among 500 repetitions. The right subplot is for ${\mathcal {P}}_a$ among 500 repetitions.

**Figure 4:**
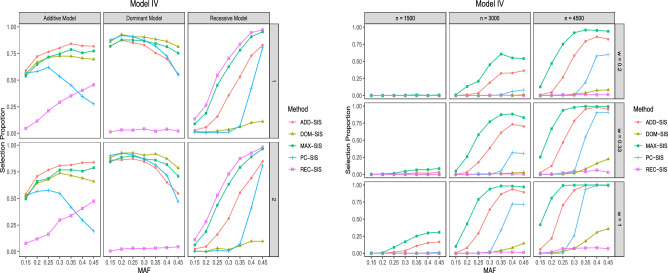
Selection proportions of different methods in Model IV. The left subplot is for ${\mathcal {P}}^k_s, k = 1,2,\ldots ,6$ among 500 repetitions, when sample size is 3000 and case-to-control ratio is 0.2. The right subplot is for ${\mathcal {P}}_a$ among 500 repetitions.

In this section, we conduct simulation studies to assess the performances of REC-SIS, ADD-SIS, DOM-SIS and MAX-SIS by comparing with PC-SIS^19^.

For each genetic model, the dimension of SNPs is $$m = 10^5$$. Since the sample size, the case-to-control ratio and the minor allelic frequency (MAF)^20^ can affect the association analysis in a case-control study, we consider different settings on them. To be specific, we choose the sample size *n* from $$\{1500,3000,4500\}$$, the case-to-control ratio $$ w = p:q$$ from $$\{1,1/3,1/5\}$$ and MAF $$\alpha $$ from $$ \{0.15,0.20,0.25,0.30,0.35,0.40,0.45\}$$. Because only the counts of genotypes are needed to calculate the statistics of interest, there is no need to generate original samples $$\{({\varvec{g}}_l,y_l), l = 1, 2,\cdots ,n\}$$ in the simulation studies. Instead, we can just generate the count data from the trinomial distribution for each dataset. For the *k*th genetic variant (SNP), the count vector of three genotypes for case samples $$(r_{0k},r_{1k},r_{2k})$$ follows the trinomial distribution $$\textrm{Mul}(np,p_{0k}/p,p_{1k}/p,p_{2k}/p)$$ and that for control samples $$(s_{0k},s_{1k},s_{2k})$$ follows the trinomial distribution $$\textrm{Mul}(nq,q_{0k}/q,q_{1k}/q,q_{2k}/q)$$, where $$p_{0k}+p_{1k}+p_{2k} = p,~q_{0k}+q_{1k}+q_{2k}=q$$.

In each dataset, the first six SNPs are set to be related with *Y* and the rest SNPs are independent of *Y*. For the control sample, the count vector of each SNP $G_k (k \in \{1,2, \ldots , 10^5\})$ $(s_{0k},s_{1k},s_{2k})$ is generated from the trinomial distribution $\textrm{Mul}(nq,q_{0k}/q,q_{1k}/q,q_{2k}/q)$, where $q_{0k} = q(1-\alpha )^2, q_{1k} = 2q\alpha (1-\alpha ), q_{2k} = q\alpha ^2$ with $\alpha$ being the MAF. For the case sample, the count vector of each irrelevant SNP $G_k, ~(k \in \{7,8, \ldots , 10^5\})$ $(r_{0k},r_{1k},r_{2k})$ is generated from $\textrm{Mul}(np,p_{0k}/p,p_{1k}/p,p_{2k}/p)$ with $p_{ik}/p = q_{ik}/q, i = 0,1,2$; while the count vector for each relevant SNP $G_k (k \in \{1,2, \ldots , 6\})$ $(r_{0k},r_{1k},r_{2k})$ is generated from the trinomial distribution $\textrm{Mul}(np,p_{0k}/p,p_{1k}/p,p_{2k}/p)$, where $(p_{0k},p_{1k},p_{2k})$ are functions of $(q_{0k},q_{1k},q_{2k})$ and are diverse for different genetic models. Four different genetic models are considered, that is, recessive genetic model, additive genetic model, dominant genetic model and mixture of them, which are denoted as Model I, Model II, Model III and Model IV as follows, respectively.

Under each genetic model, 500 repetitions are conducted to compare the performances of different methods. We employ two criteria to measure the effectiveness of each screening approach. One is the proportion for each relevant SNP $G_k, k \in {\mathscr {A}}$ that is selected among all the 500 repetitions and is denoted as ${\mathcal {P}}^k_s$. The other is the proportion that all the relevant SNPs are simultaneous selected among these 500 repetitions, which is denoted as ${\mathcal {P}}_a$.

Model I. Data are generated from the recessive genetic model. For the relevant SNPs $G_k, (k=1,2,\ldots ,6)$, $p_{0k} = \frac{pq_{0k}}{q_{0k}+q_{1k}+\lambda q_{2k}},p_{1k} = \frac{pq_{1k}}{q_{0k}+q_{1k}+\lambda q_{2k}},p_{2k} = \frac{p\lambda q_{2k}}{q_{0k}+q_{1k}+\lambda q_{2k}}$, with $\lambda =1.8$.
Model II. Data are generated from the additive genetic model. For the relevant SNPs $G_k, (k=1,2,\ldots ,6)$, $p_{0k} = \frac{pq_{0k}}{q_{0k}+\lambda q_{1k}+(2\lambda -1) q_{2k}},p_{1k} = \frac{p\lambda q_{1k}}{q_{0k}+\lambda q_{1k}+(2\lambda -1) q_{2k}},p_{2k} = \frac{p(2\lambda -1)q_{2k}}{q_{0k}+\lambda q_{1k}+(2\lambda -1) q_{2k}}$, with $\lambda =1.4$.
Model III. Data are generated from the dominant genetic model. For the relevant SNPs $G_k, (k=1,2,\ldots ,6)$, $p_{0k} = \frac{pq_{0k}}{q_{0k}+\lambda q_{1k}+\lambda q_{2k}},p_{1k} = \frac{p\lambda q_{1k}}{q_{0k}+\lambda q_{1k}+\lambda q_{2k}},p_{2k} = \frac{p\lambda q_{2k}}{q_{0k}+\lambda q_{1k}+\lambda q_{2k}}$, with $\lambda =1.6$.
Model IV. Data are generated from the mixture of three genetic models. Relevant SNPs $G_1$ and $G_2$ are generated as those in Model I, relevant SNPs $G_3$ and $G_4$ are generated as those in Model II and relevant SNPs $G_5$ and $G_6$ are generated as those in Model III.

**Figure 5:**
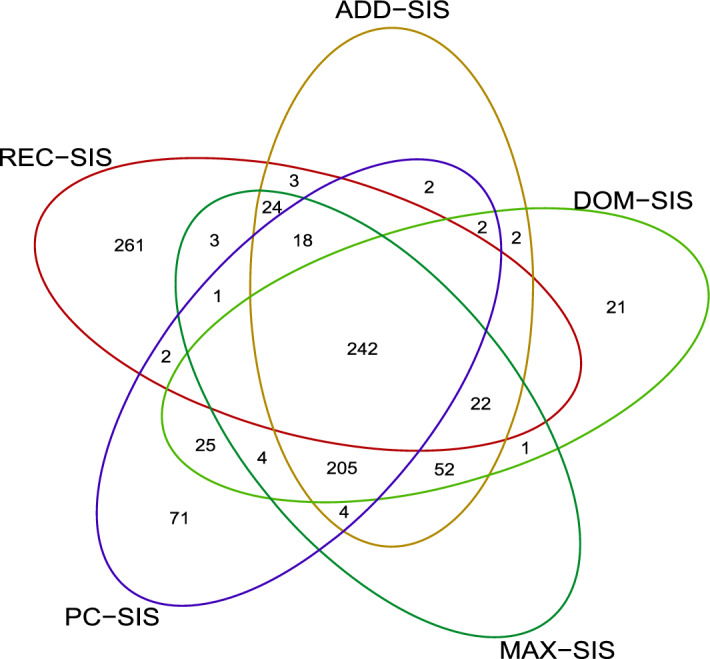
The venn diagram for the results of all the five procedures.

For each model, the proportions ${\mathcal {P}}^k_s, k = 1,2,\ldots ,6$ and ${\mathcal {P}}_a$ are calculated with the constant $d = [n/ \log n]$, where [*a*] denotes the integer part of *a*. The results are plotted in Figs. 1, 2, 3 and 5. Since in Models I, II and III, the first six relevant SNPs are generated from the same distribution, ${\mathcal {P}}^k_s, k = 1, 2, \ldots 6$ are similar in these models. Therefore, we only plot the results for ${\mathcal {P}}^1_s$ in Figs. 1, 2 and 3. In Model IV, the relevant SNPs are generated from different genetic models, so the results for ${\mathcal {P}}^k_s, k = 1, 2, \ldots 6$ are plotted in Fig. 3. Besides, the results for ${\mathcal {P}}_a$ are all plotted in Figs. 1, 2, 3 and 5.

Results in Fig. 1 correspond to the recessive genetic model. It can be seen that REC-SIS performs the best, MAX-SIS comes the second, and DOM-SIS is the worst. As Fig. 1 illuminates, the ability of detecting $G_1$ for all the screening approaches increases as sample size, the case-to-control ratio and MAF increase. In addition, it shows that PC-SIS almost fails to detect the relevant SNPs when MAF is less than 0.3.

The simulation results for Model II are presented in Fig. 2. It shows that when the underlying genetic model is exactly additive genetic model, ADD-SIS performs best, MAX-SIS ranks the second. As Fig. 2 displays, the ability to detect relevant SNPs for all the screening approaches increases as the sample size and the case-to-control ratio increase. REC-SIS has low powers when MAF is small. The detection proportions of DOM-SIS first increase and then decrease slightly as MAF increases. In general, the detection proportions of MAX-SIS and ADD-SIS increase as MAF becomes larger. Whereas, the detection proportions of PC-SIS first increase slightly and then decrease dramatically as MAF increases.

The results for Model III are exhibited in Fig. 3. It shows that when the underlying genetic model is exactly dominant genetic model, DOM-SIS performs the best and REC-SIS can hardly work. As shown in Fig. 3 , the ability of detecting $G_1$ for all the screening approaches increases as the sample size and the case-to-control ratio increase. Furthermore, when MAF is greater than 0.25, the detection proportions for all the methods except REC-SIS decline as MAF increases. From the right subplot of Fig. 3, we can see that the performances of ADD-SIS and PC-SIS are greatly influenced by MAF, while those of DOM-SIS and MAX-SIS are robust against MAF.

As for Model IV, since the effects of sample size and case-to-control ratio on the performances of different methods have been demonstrated in the above three models, we take $n = 3000, w = 0.2$ as representative to demonstrate the effects of different genetic models. The results of ${\mathcal {P}}_s^{k}, k =1,2,\ldots ,6$ when $n = 3000, w = 0.2$ are illustrated in the left subplot of Fig. 4 and the results of ${\mathcal {P}}_a$ under all scenarios are shown in the right subplot of Fig. 4 . Since the six relevant SNPs follow different genetic models, REC-SIS, ADD-SIS and DOM-SIS can not excel MAX-SIS and PC-SIS uniformly for all the relevant SNPs. Consistent with the results shown before, REC-SIS has the highest detection proportion for $G_1$ and $G_2$, ADD-SIS has the highest detection proportion for $G_3$ and $G_4$, and DOM-SIS has the highest detection proportion for $G_5$ and $G_6$. None of REC-SIS, ADD-SIS and DOM-SIS has the best performance uniformly. However, no matter what the underlying genetic relationship is, MAX-SIS always has excellent performance. As for ${\mathcal {P}}_a$, MAX-SIS outperforms all the other methods significantly.

From the simulation results above, we can see that sample size and case-to-control ratio are two important factors that affect the association analysis. It is rational that increasement in sample size can enhance the efficiency in identifying associated SNPs. As for case-to-control ratio, when the ratio approaches 1, all the methods have better performances than conditions with larger ratios. Given the size for case sample, increasing the size for control sample has little contribution on the performances of all the methods. For example, when $w = p:q = 1/3, n =3000$ and $w= p:q = 1:5, n =4500$, that is when the case sample size $r = 750$, and the control sample size $s = 2250$ and 3750 respectively, the selection proportions of all the five screening methods have similar results no matter how MAF varies. The effect of MAF is not monotonic. In recessive model, the selection proportions of all the five methods increase as MAF increases. However, in other models, the selection proportions of some methods first increase and later decrease as MAF increases. Under all the scenarios considered, MAX-SIS is the most robust method among these five screening methods.

Overall, we can come to the conclusion that if all the candidate SNPs follow the same known genetic model, one of REC-SIS, ADD-SIS, DOM-SIS performs the best. However, the genetic model is always complicated and unknown in practice. In this case, MAX-SIS is recommended to reach robustness and efficiency.

## Application to a real dataset

We apply the proposed screening procedures to a real case-control data of type 1 diabetes for British people^1^. The data contains 459,446 SNPs for 2938 controls and 1963 cases. Since there exist some missing values in the genotype data, the number of observed genotypes for a single SNP varies across all the SNPs. Count the number of missing values for each partially observed SNP. And it shows that the average number and the largest number of all these counts is 16.72 and 503, respectively, and the $25\%, 50\%, 75\%$ quantile of these counts are 4, 7 and 13, respectively. To make aCATT and aMAX statistics have similar consistency rates for all the SNPs, SNPs with missing ratio large than 1% are deleted. Besides, SNPs with only two genotypes being observed are also removed from the dataset, resulting in 352,659 SNPs to be analyzed. For each SNP, the allele with lower frequency is treated as the risk allele. We use REC-SIS, ADD-SIS, DOM-SIS, MAX-SIS and PC-SIS to screen out the redundant SNPs, with the parameter *d* being $[4901/\log (4901)] = 576.$ The results are shown in the venn diagram in Fig. 5 to display the screening results of all the five procedures. It shows that 242 SNPs are selected by all the procedures. Among these SNPs, SNPs rs9272346 and rs9272346 have been reported to be associated with type 1 diabetes^1^. This indicates that there may be some important association information contained in these SNPs which need to be further investigated. We list these 242 SNPs in Table 3.

**Table 3:** The 242 SNPs selected by all the five screening procedures.

rs1113523	rs203888	rs12527415	rs3130532	rs2523467	rs574710	rs9268645	rs7768538
rs1217200	rs9393881	rs11758688	rs2248880	rs2395034	rs539703	rs3135393	rs7453920
rs1230666	rs203884	rs3094123	rs6906846	rs3131631	rs3132959	rs1051336	rs6902723
rs1230658	rs1233708	rs3130649	rs2524067	rs2736177	rs926591	rs9268831	rs6903130
rs1230649	rs406511	rs3095350	rs7382297	rs1046089	rs3129900	rs9268877	rs9296044
rs6679677	rs2523443	rs6924600	rs2523537	rs760293	rs4959093	rs9270986	rs2857212
rs1217396	rs1611350	rs6457282	rs2523535	rs3130048	rs910050	rs615672	rs2071474
rs2488457	rs1736913	rs9295931	rs2523534	rs805301	rs910049	rs9271208	rs241427
rs9467704	rs2517646	rs2074508	rs2596437	rs707918	rs3129932	rs3129768	rs241403
rs9379851	rs3130391	rs3218815	rs5025315	rs376510	rs3129934	rs9272219	rs3101942
rs6933583	rs3094055	rs2074512	rs5022119	rs805292	rs9268403	rs9272346	rs241400
rs4712980	rs1012411	rs4711247	rs2523638	rs707915	rs12201454	rs9272723	rs151719
rs9393708	rs3132644	rs3132581	rs3997982	rs3130484	rs2894254	rs9273363	rs3129304
rs9358932	rs3132636	rs2530710	rs2596571	rs3131379	rs3129953	rs9275134	rs3129303
rs9379855	rs3129818	rs1634717	rs2523485	rs480092	rs2076533	rs2856688	rs10947374
rs9393713	rs3129819	rs1634718	rs3099849	rs2763979	rs9268480	rs7775228	rs9296069
rs1977	rs970269	rs1632854	rs2507976	rs550513	rs3763307	rs9469220	rs13215059
rs10456045	rs3132625	rs2844670	rs9266774	rs406936	rs6930933	rs2647015	rs13215062
rs9358945	rs3094050	rs2523865	rs9266775	rs3130287	rs2001099	rs2858308	rs376877
rs7763910	rs3094703	rs3130544	rs4081552	rs1150753	rs2001097	rs9275418	rs2179920
rs7773938	rs3094045	rs9405050	rs2596517	rs204991	rs3135378	rs9275523	rs3117242
rs4634439	rs3094034	rs1265052	rs2596464	rs204990	rs3135377	rs6936863	rs3128923
rs12190473	rs3130112	rs3130975	rs3131622	rs2071278	rs3135376	rs3916765	rs3117230
rs201002	rs3130113	rs3095324	rs2844507	rs3131294	rs2395161	rs9461799	rs872956
rs200995	rs3094694	rs13200022	rs2244579	rs377763	rs2395164	rs2227127	rs2395351
rs200991	rs8233	rs3130564	rs2248459	rs3134926	rs2395167	rs9276429	rs3116985
rs149946	rs3095329	rs2106074	rs2248462	rs424232	rs2213580	rs9276431	rs3129248
rs149969	rs3094127	rs887464	rs2248617	rs3130311	rs3135366	rs9276432	rs10807124
rs149970	rs3094122	rs1265181	rs3099844	rs1265777	rs9268557	rs9276440	rs11171739
rs202906	rs10947091	rs12199773	rs2516422	rs9268230	rs9268560	rs9276490	rs1265566
rs17696736	rs9746695						

## Conclusion

Screening SNPs in case-control study is a commonly encountered task in modern biomedical research. And CATT and MAX statistics are the most widely used screening measures for this issue. However, the theoretical guarantees for the application of CATT and MAX to SNP screening have not been investigated. We fill this gap by adjusting CATTs and MAX test, and proposing screening procedures based on the adjusted statistics. Sure screening properties and ranking consistency properties of these screening procedures are proved. Simulation results show that when the underlying genetic model is unknown, which is often the case in practice, MAX-SIS performs the best.

Despite of the high efficiency of the proposed procedures, there exist some factors that affect their performances. First, numerical simulations show that when both MAF and sample size are small, REC-SIS, ADD-SIS, DOM-SIS, MAX-SIS and PC-SIS all perform badly. This is because that under this situation, the number of samples possessed with minor alleles is too small to provide enough information for the association analysis. Second, it is obviously that the value of the parameter *d* influence the performances of different methods. We determine the value of *d* based on works in the previous literatures. Since how to choose an optimal *d* is not the focus of this work, we will conduct more detailed analysis further. Third, when there exist covariates to be adjusted for, new procedures need to be developed, which will be studied in a future work.

## Supplementary Information

Supplementary Information.

## Data Availability

All data included in this study are available upon request by contacting with the corresponding author. To facilitate the usage for the proposed methods, the codes are available upon request by contacting with the corresponding author.
