# Supplementary material for: Screening properties of trend tests in genetic association studies
Source: Sci Rep. 2023 Jun 5;13:9139. doi: 10.1038/s41598-023-35929-4 (PMC10241885; doi:10.1038/s41598-023-35929-4)
Supplement: Supplementary file 1 — Supplementary Information. [file 41598_2023_35929_MOESM1_ESM.pdf]

Supplemental Materials for "Screening properties of trend tests in genetic association studies"

Theoretical Proofs

Proofs for theorems in this work are presented here. First, we display a lemma as follows.

Lemma 1 (Bernstein inequality): Suppose $X_i, i = 1, 2, \cdots, N$ are independent random variables with expectations of 0. If there exists a constant M satisfying $|X_i| \leq M, a.s$, it follows that

$$P\Big(\big|\sum_{i=1}^{N} X_i\big| \geq t\Big) \leq \exp\{-\frac{3t^2}{6\sum\limits_{i=1}^{N} E(X_i^2) + 2Mt}\}. \quad (1)$$

Proof of Theorem 1

We first prove the property when $j = 0$, and the property when $j = \frac{1}{2}, 1$ can be proved in the same way.

For convenience, we denote $\alpha_{0,k} = \sum\limits_{i=0}^{2} X_{i,0}(qp_{ik} - pq_{ik})$, $\hat{\alpha}_{0,k} = \sum\limits_{i=0}^{2} X_{i,0}(\hat{q}\hat{p}_{ik} - \hat{p}\hat{q}_{ik})$, and $\beta_{0,k} = \Big(pq\big[\sum\limits_{i=0}^{2} X_{i,0}^2 f_{ik} - (\sum\limits_{i=0}^{2} X_{i,0} f_{ik})^2\big]\Big)^{\frac{1}{2}}$, $\hat{\beta}_{0,k} = \Big(\hat{p}\hat{q}\big[\sum\limits_{i=0}^{2} X_{i,0}^2 \hat{f}_{ik} - (\sum\limits_{i=0}^{2} X_{i,0} \hat{f}_{ik})^2\big]\Big)^{\frac{1}{2}}$. Then we have $\omega_{0,k} = \frac{\alpha_{0,k}}{\beta_{0,k}}$, $\hat{\omega}_{0,k} = \frac{\hat{\alpha}_{0,k}}{\hat{\beta}_{0,k}}, k = 1, 2, \cdots, m$.

Firstly, we draw a conclusion that

$$\frac{\hat{\beta}_{0,k}}{\beta_{0,k}} = 1 + O_p(n^{-\frac{1}{4}}). \quad (2)$$

Since the samples are independent identically distributed, by central limit theorem, we have $\sqrt{n}(\hat{p} - p) \xrightarrow{d} N\big(0, p(1-p)\big)$, $\sqrt{n}(\hat{q} - q) \xrightarrow{d} N\big(0, q(1-q)\big)$, and $\sqrt{n}(\hat{f}_{ik} - f_{ik}) \xrightarrow{d} N\big(0, f_{ik}(1-f_{ik})\big)$, for $i = 0, 1, 2$ and $k = 1, 2, \cdots, m$. Hence, we gain that $\hat{p} = p + O_p(\frac{1}{\sqrt{n}})$, $\hat{q} - q = O_p(\frac{1}{\sqrt{n}})$, and $\hat{f}_{ik} = f_{ik} + O_p(\frac{1}{\sqrt{n}})$, for $i = 0, 1, 2$ and $k = 1, 2, \cdots, m$.

Therefore, we derive that

$$\begin{aligned}\hat{\beta}_{0,k}^2 &= \hat{p}\hat{q}\Big[\sum_{i=0}^{2} X_{i,0}^2 \hat{f}_{ik} - \big(\sum_{i=0}^{2} X_{i,0}\hat{f}_{ik}\big)^2\Big] \\ &= \big(p + O_p(\tfrac{1}{\sqrt{n}})\big)\big(q + O_p(\tfrac{1}{\sqrt{n}})\big) \\ &\quad \times\Big[\sum_{i=0}^{2} X_{i,0}^2\big[f_{ik} + O_p(\tfrac{1}{\sqrt{n}})\big] - \big(\sum_{i=0}^{2} X_{i,0}\big[f_{ik} + O_p(\tfrac{1}{\sqrt{n}})\big]\big)^2\Big] \\ &= \big(pq + O_p(\tfrac{1}{\sqrt{n}})\big)\Big[\sum_{i=0}^{2} X_{i,0}^2 f_{ik} - \big(\sum_{i=0}^{2} X_{i,0} f_{ik}\big)^2 + O_p(\tfrac{1}{\sqrt{n}})\Big] \\ &= pq\Big[\sum_{i=0}^{2} X_{i,0}^2 f_{ik} - \big(\sum_{i=0}^{2} X_{i,0} f_{ik}\big)^2\Big] + O_p(\tfrac{1}{\sqrt{n}}) \\ &= \beta_{0,k}^2 + O_p(\tfrac{1}{\sqrt{n}}).\end{aligned} \tag{3}$$

Immediately, the equation (2) can be deduced by the above equation (3).

Secondly, we demonstrate that

$$P\big(|\hat{\omega}_{0,k} - \omega_{0,k}| \geq c_0 n^{-\tau}\big) = P\big(\big|\hat{\alpha}_{0,k} - \alpha_{0,k}\big| \geq c_1 n^{-\tau} + c_2 n^{-\frac{1}{4}}\big), \tag{4}$$

where c_1 and c_2 are constants. Since $\hat{\alpha}_{0,k},\ \hat{\beta}_{0,k},\ \alpha_{0,k},\ \beta_{0,k}$ are bounded, we obtain that

$$\begin{aligned}& P\big(|\hat{\omega}_{0,k} - \omega_{0,k}| \geq c_0 n^{-\tau}\big) \\ = \;& P\big(\big|\tfrac{\hat{\alpha}_{0,k}}{\hat{\beta}_{0,k}} - \tfrac{\alpha_{0,k}}{\beta_{0,k}}\big| \geq c_0 n^{-\tau}\big) \\ = \;& P\big(\big|\hat{\alpha}_{0,k}\big(1 + O_p(n^{-\frac{1}{4}})\big) - \alpha_{0,k}\big| \geq \beta_{0,k} \cdot c_0 n^{-\tau}\big) \\ = \;& P\big(\big|\hat{\alpha}_{0,k} - \alpha_{0,k}\big| \geq \beta_{0,k} \cdot c n^{-\tau} + \hat{\alpha}_{0,k} O_p(n^{-\frac{1}{4}})\big) \\ = \;& P\big(\big|\hat{\alpha}_{0,k} - \alpha_{0,k}\big| \geq c_1 n^{-\tau} + c_2 n^{-\frac{1}{4}}\big),\end{aligned}$$

where c_1 and c_2 are two constants. Hence, equation (4) is proved.

Thirdly, we deduce the inequality of

$$P\big(|\hat{\alpha}_{0,k} - \alpha_{0,k})| \geq \epsilon\big) \leq 12 \exp\big\{ - \frac{n\epsilon^2}{72\zeta_{max}^2 + 8\epsilon\zeta_{max}}\big\}, \tag{5}$$

where ζ_{max} is defined in Condition (C1). Let $u_{l,ik} = I(Y_l = 1, X_{lk} = i)$, for $l = 1, 2, \cdots, n$. Because $\{u_{l,ik}\}_{l=1}^{n} \overset{iid}{\sim}$ Bernoulli($\mathrm{p_{ik}}$), we know that $|u_{l,ik} - p_{ik}| \leq 1, E(u_{l,ik} - p_{ik})^2 \leq \frac{1}{4}$. By Bernstein inequality, we have

$$\begin{aligned}P\big(\tfrac{1}{n}\big|\sum_{l=1}^{n}(u_{l,ik} - p_{ik})\big| \geq t\big) &= P\big(\big|\sum_{l=1}^{n}(u_{l,ik} - p_{ik})\big| \geq nt\big) \\ &\leq \exp\big\{ - \tfrac{3n^2t^2}{6np_{ik}(1-p_{ik}) + 2nt}\big\} \\ &\leq \exp\big\{ - \tfrac{6nt^2}{3+4t}\big\}.\end{aligned}$$

Note that $\hat{p}_{ik} = \frac{1}{n}\sum_{l=1}^{n} u_{l,ik}$. Consequently, we have

$$P\big(|\hat{p}_{ik} - p_{ik}| \geq t\big) \leq \exp\{-\frac{6nt^2}{3+4t}\},\ k = 1, 2\cdots, m;\ i = 0, 1, 2. \quad (6)$$

Analogously, we also obtain

$$P\big(|\hat{q}_{ik} - q_{ik}| \geq t\big) \leq \exp\{-\frac{6nt^2}{3+4t}\},\ k = 1, 2\cdots, m;\ i = 0, 1, 2. \quad (7)$$

Let $u_l = I(Y_l = 1)$, for $l = 1, 2, \cdots, n$. Because $\{u_l\}_{l=1}^{n} \overset{iid}{\sim}$ Bernoulli(p), we know that $|u_l - p| \leq 1, E(u_l - p)^2 \leq \frac{1}{4}$. By Bernstein inequality, we have

$$\begin{aligned} P\big(\tfrac{1}{n}|\sum_{l=1}^{n}(u_l - p)| \geq t\big) &= P\big(|\sum_{l=1}^{n}(u_l - p)| \geq nt\big) \\ &\leq \exp\big\{-\tfrac{3n^2t^2}{6np(1-p)+2nt}\big\} \\ &\leq \exp\big\{-\tfrac{6nt^2}{3+4t}\big\}. \end{aligned}$$

Therefore, we conclude that

$$P\big(|\hat{p} - p)| \geq t\big) \leq \exp\{-\frac{6nt^2}{3+4t}\}. \quad (8)$$

Analogously, we also obtain that

$$P\big(|\hat{q} - q)| \geq t\big) \leq \exp\{-\frac{6nt^2}{3+4t}\}. \quad (9)$$

$$
\begin{aligned}
& P\Big(\big|\hat{\alpha}_{0,k}-\alpha_{0,k})\big| \geq \epsilon\Big) \\
= \; & P\Big(\big|\sum_{i=0}^{2} X_{i,0}(\hat{q}\hat{p}_{ik}-\hat{p}\hat{q}_{ik}) - \sum_{i=0}^{2} X_{i,0}(qp_{ik}-pq_{ik}))\big| \geq \epsilon\Big) \\
= \; & P\Big(\Big|\sum_{i=0}^{2}\big[X_{i,0}\hat{q}(\hat{p}_{ik}-p_{ik}) - X_{i,0}\hat{p}(\hat{q}_{ik}-q_{ik}) + X_{i,j}p_{ik}(\hat{q}-q) - X_{i,j}q_{ik}(\hat{p}-p)\big]\Big| \geq \epsilon\Big) \\
\leq \; & \sum_{i=0}^{2} P\Big(\big|X_{i,0}\hat{q}(\hat{p}_{ik}-p_{ik})\big| \geq \tfrac{\epsilon}{12}\Big) + \sum_{i=0}^{2} P\Big(X_{i,0}\big|\hat{p}(\hat{q}_{ik}-q_{ik})\big| \geq \tfrac{\epsilon}{12}\Big) \\
& + \sum_{i=0}^{2} P\Big(X_{i,0}\big|p_{ik}(\hat{q}-q)\big| \geq \tfrac{\epsilon}{12}\Big) + \sum_{i=0}^{2} P\Big(X_{i,0}\big|q_{ik}(\hat{p}-p)\big| \geq \tfrac{\epsilon}{12}\Big) \qquad \text{for } X_{i,0} \in \{0, \tfrac{1}{2}, 1\} \\
\leq \; & \sum_{i=0}^{2}\Big[P\big(|\hat{p}_{ik}-p_{ik}| \geq \tfrac{\epsilon}{12\zeta_{max}}, \hat{q} \leq \zeta_{max}\big) + P(\hat{q} > \zeta_{max})\Big] \qquad \text{by Condition 1} \\
& + \sum_{i=0}^{2}\Big[P\big(|\hat{q}_{ik}-q_{ik}| \geq \tfrac{\epsilon}{12\zeta_{max}}, \hat{p} \leq \zeta_{max}\big) + P(\hat{p} > \zeta_{max})\Big] \\
& + \sum_{i=0}^{2} P\big(|\hat{q}-q| \geq \tfrac{\epsilon}{12\zeta_{max}}\big) + \sum_{i=0}^{2} P\big(|\hat{p}-p| \geq \tfrac{\epsilon}{12\zeta_{max}}\big) \\
\leq \; & \sum_{i=0}^{2} P\big(|\hat{p}_{ik}-p_{ik}| \geq \tfrac{\epsilon}{12\zeta_{max}}\big) + 3P\big(|\hat{q}-q| \geq \tfrac{\epsilon}{12\zeta_{max}}\big) \\
& + \sum_{i=0}^{2} P\big(|\hat{q}_{ik}-q_{ik}| \geq \tfrac{\epsilon}{12\zeta_{max}}\big) + 3P\big(|\hat{p}-p| \geq \tfrac{\epsilon}{12\zeta_{max}}\big) \\
& +3P(\hat{p} > \zeta_{max}) + 3P(\hat{q} > \zeta_{max})
\end{aligned} \tag{10}
$$

When n is sufficiently large, by Condition (C1), we have

$$
P(\hat{p} > \zeta_{max}) = P\big(p > \zeta_{max} + O_p(n^{-\frac{1}{2}})\big) = 0. \tag{11}
$$

Similarly, when n is sufficiently large,

$$
P(\hat{q} > \zeta_{max}) = P\big(q > \zeta_{max} + O_p(n^{-\frac{1}{2}})\big) = 0. \tag{12}
$$

By inequality (6), for $i = 0, 1, 2$, we have

$$
P\big(|\hat{p}_{ik}-p_{ik}| \geq \frac{\epsilon}{12\zeta_{max}}\big) \leq \exp\{-\frac{6n\frac{\epsilon^2}{144\zeta_{max}^2}}{3+4\frac{\epsilon}{12\zeta_{max}}}\} = \exp\{-\frac{n\epsilon^2}{72\zeta_{max}^2 + 8\zeta_{max}\epsilon}\}. \tag{13}
$$

By inequality (7), for $i = 0, 1, 2$, we have

$$
P\big(|\hat{q}_{ik}-q_{ik}| \geq \frac{\epsilon}{12\zeta_{max}}\big) \leq \exp\{-\frac{6n\frac{\epsilon^2}{144\zeta_{max}^2}}{3+4\frac{\epsilon}{12\zeta_{max}}}\} = \exp\{-\frac{n\epsilon^2}{72\zeta_{max}^2 + 8\zeta_{max}\epsilon}\}. \tag{14}
$$

By inequality (8), we have

$$
P\big(|\hat{p}-p| \geq \frac{\epsilon}{12\zeta_{max}}\big) \leq \exp\{-\frac{6n\frac{\epsilon^2}{144\zeta_{max}^2}}{3+4\frac{\epsilon}{12\zeta_{max}}}\} = \exp\{-\frac{n\epsilon^2}{72\zeta_{max}^2 + 8\zeta_{max}\epsilon}\}. \tag{15}
$$

By inequality (9), we have

$$P\big(|\hat{q}-q| \geq \frac{\epsilon}{12\zeta_{max}}\big) \leq \exp\{-\frac{6n\frac{\epsilon^2}{144\zeta_{max}^2}}{3+4\frac{\epsilon}{12\zeta_{max}}}\} = \exp\{-\frac{n\epsilon^2}{72\zeta_{max}^2+8\zeta_{max}\epsilon}\}. \tag{16}$$

Combining inequalities (10)-(16), we derive the inequality (5).

Combining inequalities (4) and (5), we prove

$$P\big(|\hat{\omega}_{0,k}-\omega_{0,k}| \geq c_0 n^{-\tau}\big) \leq O\big(\exp\{-(c_1 n^{1-2\tau}+c_2 n^{\frac{1}{2}})\}\big), \tag{17}$$

where the constants $c_1 > 0$ and $c_2 > 0$ are not necessarily the same as those in inequality (4). Furthermore, according to inequality (17), we can conclude the first part of Theorem 1,

$$\begin{aligned} P\big(\max_{1\leq k\leq m} |\hat{\omega}_{0,k}-\omega_{0,k}| \geq c_0 n^{-\tau}\big) &\leq m \max_{1\leq k\leq m} P\big(|\hat{\omega}_{0,k}-\omega_{0,k}| \geq c_0 n^{-\tau}\big) \\ &= O\big(m\exp\{-(c_1 n^{1-2\tau}+c_2 n^{\frac{1}{2}})\}\big), \end{aligned}$$

where the constants $c_1 > 0$ and $c_2 > 0$ are different from those in inequalities (4) and (17).

Lastly, for the second part of Theorem 1,

$$\begin{aligned} P\big(\mathscr{A}_0^* \subseteq \hat{\mathscr{A}}_0^*\big) &\geq P\big(k \in \mathscr{A}_0^*, |\hat{\omega}_{0,k}-\omega_{0,k}| < c_0 n^{-\tau}\big) \\ &= P\big(\max_{k\in\mathscr{A}_0^*} |\hat{\omega}_{0,k}-\omega_{0,k}| < c_0 n^{-\tau}\big) \\ &= 1 - P\big(\min_{k\in\mathscr{A}_0^*} |\hat{\omega}_{0,k}-\omega_{0,k}| \geq c_0 n^{-\tau}\big) \\ &\geq 1-\kappa \max_{1\leq k\leq m} P\big(|\hat{\omega}_{0,k}-\omega_{0,k}| \geq c_0 n^{-\tau}\big) \\ &\geq 1 - O(\kappa\exp\{-(c_1 n^{1-2\tau}+c_2 n^{\frac{1}{2}})\}), \end{aligned} \tag{18}$$

where κ is the cardinality of $\mathscr{A}_0^*$, $c_1 > 0$ and $c_2 > 0$ are the same as those in Theorem 1 (ii).

Proof of Theorem 2

$$\begin{aligned} & P\big(|\hat{\nu}_k-\nu_k| \geq c_0 n^{-\tau}\big) \\ = & \sum_{j\in\{0,\frac{1}{2},1\}} P\big(\big||\hat{\omega}_{j,k}|-|\omega_{j,k}|\big| \geq c_0 n^{-\tau}, |\omega_{j,k}| = \max_{j_1\in\{0,\frac{1}{2},1\}}\{|\omega_{j_1,k}|\}, |\hat{\omega}_{j,k}| = \max_{j_1\in\{0,\frac{1}{2},1\}}\{|\hat{\omega}_{j_1,k}|\}\big) \\ & + \sum_{\substack{j\neq j_1 \\ j,j_1\in\{0,\frac{1}{2},1\}}} P\big(\big||\hat{\omega}_{j,k}|-|\omega_{j_1,k}|\big| \geq c_0 n^{-\tau}, \omega_{j,k} = \max_{j_2\in\{0,\frac{1}{2},1\}}\{|\omega_{j_2,k}|\}, \hat{\omega}_{j_1,k} = \max_{j_2\in\{0,\frac{1}{2},1\}}\{|\hat{\omega}_{j_2,k}|\}\big) \\ \leq & \sum_{j\in\{0,\frac{1}{2},1\}} P\big(\big||\hat{\omega}_{j,k}|-|\omega_{j,k}|\big| \geq c_0 n^{-\tau}\big) + \sum_{\substack{j\neq j_1 \\ j,j_1\in\{0,\frac{1}{2},1\}}} P\big(|\hat{\omega}_{j,k}| > |\hat{\omega}_{j_1,k}|, |\omega_{j,k}| < |\omega_{j_1,k}|\big) \\ \leq & \sum_{j\in\{0,\frac{1}{2},1\}} P\big(\big|\hat{\omega}_{j,k}-\omega_{j,k}\big| \geq c_0 n^{-\tau}\big) + \sum_{\substack{j\neq j_1 \\ j,j_1\in\{0,\frac{1}{2},1\}}} P\big(|\hat{\omega}_{j,k}| > |\hat{\omega}_{j_1,k}|, |\omega_{j,k}| < |\omega_{j_1,k}|\big). \end{aligned} \tag{19}$$

For the second term of inequality (19),

$$
\begin{aligned}
& P\big(|\hat{\omega}_{j,k}| > |\hat{\omega}_{j_1,k}|, |\omega_{j,k}| < |\omega_{j_1,k}|\big) \\
\leq\; & P\big(|\hat{\omega}_{j,k}| > |\hat{\omega}_{j_1,k}|, |\omega_{j,k}| < |\omega_{j_1,k}|, |\hat{\omega}_{j,k} - \omega_{j,k}| < c_0 n^{-\tau}, |\hat{\omega}_{j_1,k} - \omega_{j_1,k}| < c_0 n^{-\tau}\big) \\
& + P\big(|\hat{\omega}_{j,k} - \omega_{j,k}| \geq c_0 n^{-\tau}\big) + P\big(|\hat{\omega}_{j_1,k} - \omega_{j_1,k}| \geq c_0 n^{-\tau}\big) \\
\leq\; & P\big(|\omega_{j,k}| < |\omega_{j_1,k}|, |\omega_{j,k}| + c_0 n^{-\tau} > |\omega_{j_1,k}| - c_0 n^{-\tau}\big) \\
& + P\big(|\hat{\omega}_{j,k} - \omega_{j,k}| \geq c_0 n^{-\tau}\big) + P\big(|\hat{\omega}_{j_1,k} - \omega_{j_1,k}| \geq c_0 n^{-\tau}\big) \\
=\; & P\big(|\omega_{j,k}| < |\omega_{j_1,k}|, |\omega_{j,k}| > |\omega_{j_1,k}| - 2c_0 n^{-\tau}\big) \\
& + P\big(|\hat{\omega}_{j,k} - \omega_{j,k}| \geq c_0 n^{-\tau}\big) + P\big(|\hat{\omega}_{j_1,k} - \omega_{j_1,k}| \geq c_0 n^{-\tau}\big) \\
=\; & O\big(\exp\{-c_1 n^{1-2\tau} - c_2 n^{\frac{1}{2}}\}\big),
\end{aligned}
\tag{20}
$$

where $c_1 > 0$ and $c_2 > 0$ are constants different from the above. The last equation holds by Theorem 1 when n is sufficiently large. Plugging inequality (20) into inequality (19) and according to Theorem 1, we can derive

$$
P\big(|\hat{\nu}_k - \nu_k| \geq c_0 n^{-\tau}\big) = O\big(\exp\{-(c_1 n^{1-2\tau} + c_2 n^{\frac{1}{2}})\}\big), \tag{21}
$$

where $c_1 > 0$ and $c_2 > 0$ are constants different from the above inequalities. Furthermore, by equation (21), we obtain

$$
\begin{aligned}
P\big(\max_{1\leq k\leq m} |\hat{\nu}_k - \nu_k| \geq c_n^{-\tau}\big) \;&\leq\; m \max_{1\leq k\leq m} P\big(|\hat{\nu}_k - \nu_k| \geq c_n^{-\tau}\big) \\
&=\; O\big(m \exp\{-(c_1 n^{1-2\tau} + c_2 n^{\frac{1}{2}})\}\big),
\end{aligned}
$$

where $c_1 > 0$ and $c_2 > 0$ are constants different from the above inequalities. Then the first part of Theorem 2 has been proven. For second part of Theorem 2,

$$
\begin{aligned}
P\big(\mathscr{A}^* \subseteq \hat{\mathscr{A}}^*\big) \;&\geq\; P\big(k \in \mathscr{A}^*, |\hat{\nu}_k - \nu_k| < c_n^{-\tau}\big) \\
&=\; P\big(\max_{k\in\mathscr{A}^*} |\hat{\nu}_k - \nu_k| < c_n^{-\tau}\big) \\
&=\; 1 - P\big(\min_{k\in\mathscr{A}^*} |\hat{\nu}_k - \nu_k| \geq c_n^{-\tau}\big) \\
&\geq\; 1 - \kappa \max_{1\leq k\leq m} P\big(|\hat{\nu}_k - \nu_k| \geq c_n^{-\tau}\big) \\
&\geq\; 1 - O(\kappa \exp\{-(c_1 n^{1-2\tau} + c_2 n^{\frac{1}{2}})\}),
\end{aligned}
\tag{22}
$$

where $c_1 > 0$ and $c_2 > 0$ are constants that are the same as those in Theorem 2 (ii).

Proof of Theorem 3

We first prove the property when $j=0$, and the property when $j=\frac{1}{2},1$ can be proved in the same way.

Define $A_n=\{\big(\min_{k\in\mathscr{A}_0^*}|\hat{\omega}_{0,k}|-\max_{k\notin\mathscr{A}_0^*}|\hat{\omega}_{0,k}|\big)\le c_0n^{-\tau}\}$.

$$\begin{aligned}
P(A_n) &\le P\big(\min_{k\in\mathscr{A}^*}(\hat{\omega}_{0,k}-\omega_{0,k})-\max_{k\notin\mathscr{A}^*}(\hat{\omega}_{0,k}-\omega_{0,k})\le -c_0n^{-\tau}\big)\\
&\le P\big(\min_{k\in\mathscr{A}^*}|\hat{\omega}_{0,k}-\omega_{0,k}|+\max_{k\notin\mathscr{A}^*}|\hat{\omega}_{0,k}-\omega_{0,k}|\ge c_0n^{-\tau}\big)\\
&\le P\big(2\max_{1\le k\le m}|\hat{\omega}_{0,k}-\omega_{0,k}|\ge c_0n^{-\tau}\big)\\
&= O\big(m\exp\{-(c_1n^{1-2\tau}+c_2n^{\frac{1}{2}})\}\big),
\end{aligned}\tag{23}$$

where c_1 and c_2 are not the same as those in Theorem 1.

$$\begin{aligned}
\sum_{n=1}^{\infty}P(A_n) &\le \sum_{n=1}^{\infty}O\big(m\exp\{-(c_1n^{1-2\tau}+c_2n^{\frac{1}{2}})\}\big)\\
&\le \sum_{n=1}^{\infty}O\big(\exp\{n^{\gamma}-c_1n^{1-2\tau}-c_2n^{\frac{1}{2}}\}\big)\\
&< \infty,
\end{aligned}\tag{24}$$

when $\gamma<\min\{\frac{1}{2},1-2\tau\}$. By the Borel Cantelli lemma, it follows that $\liminf_{n\to\infty}\big\{\min_{k\in\mathscr{A}_0^*}|\hat{\omega}_{0,k}|-\max_{k\notin\mathscr{A}_0^*}|\hat{\omega}_{0,k}|\big\}\ge 0, a.s.$ Theorem 3 has been proven.

Theorem 4 can be deduced in a similar way. Thus, we omit the procedures.
